# Blunted Myocardial Oxygenation Response During Vasodilator Stress in Patients With Hypertrophic Cardiomyopathy

**DOI:** 10.1016/j.jacc.2012.12.024

**Published:** 2013-03-19

**Authors:** Theodoros D. Karamitsos, Sairia Dass, Joseph Suttie, Emily Sever, Jacqueline Birks, Cameron J. Holloway, Matthew D. Robson, Michael Jerosch-Herold, Hugh Watkins, Stefan Neubauer

**Affiliations:** ⁎ University of Oxford Centre for Clinical Magnetic Resonance Research, Department of Cardiovascular Medicine, John Radcliffe Hospital, Oxford, United Kingdom; † University of Oxford, Centre for Statistics in Medicine, Oxford, United Kingdom; ‡ Department of Radiology, Brigham and Women's Hospital, Boston, Massachusetts

**Keywords:** athletes, cardiac magnetic resonance imaging, hypertrophy, ischemia, perfusion, BOLD, blood-oxygen level-dependent, CMR, cardiovascular magnetic resonance, ECG, electrocardiogram, HCM, hypertrophic cardiomyopathy, LGE, late gadolinium enhancement, LVH, left ventricular hypertrophy, MPRI, myocardial perfusion reserve index, PET, positron emission tomography, SI, signal intensity

## Abstract

**Objectives:**

This study sought to assess myocardial perfusion and tissue oxygenation during vasodilator stress in patients with overt hypertrophic cardiomyopathy (HCM), as well as in HCM mutation carriers without left ventricular (LV) hypertrophy, and to compare findings to those in athletes with comparable hypertrophy and normal controls.

**Background:**

Myocardial perfusion under vasodilator stress is impaired in patients with HCM. Whether this is associated with impaired myocardial oxygenation and tissue ischemia is unknown. Furthermore, it is not known whether perfusion and oxygenation are impaired in HCM mutation carriers without left ventricular hypertrophy (LVH).

**Methods:**

A total of 27 patients with overt HCM, 10 HCM mutation carriers without LVH, 11 athletes, and 20 healthy controls underwent cardiovascular magnetic resonance (CMR) scanning at 3-T. Myocardial function, perfusion (perfusion reserve index [MPRI]), and oxygenation (blood-oxygen level dependent signal intensity [SI] change) under adenosine stress were assessed.

**Results:**

MPRI was significantly reduced in HCM (1.3 ± 0.1) compared to controls (1.8 ± 0.1, p < 0.001) and athletes (2.0 ± 0.1, p < 0.001), but remained normal in HCM mutation carriers without LVH (1.7 ± 0.1; p = 0.61 vs. controls, p = 0.02 vs. overt HCM). Oxygenation response was attenuated in overt HCM (SI change 6.9 ± 1.4%) compared to controls (18.9 ± 1.4%, p < 0.0001) and athletes (18.7 ± 2.0%, p < 0.001). Interestingly, HCM mutation carriers without LVH also showed an impaired oxygenation response to adenosine (10.4 ± 2.0%; p = 0.001 vs. controls, p = 0.16 vs. overt HCM, p = 0.003 vs. athletes).

**Conclusions:**

In overt HCM, both perfusion and oxygenation are impaired during vasodilator stress. However, in HCM mutation carriers without LVH, only oxygenation is impaired. In athletes, stress perfusion and oxygenation are normal. CMR assessment of myocardial oxygenation has the potential to become a novel risk factor in HCM.

Hypertrophic cardiomyopathy (HCM) is a genetic disease with a broad spectrum of clinical manifestations and pathophysiological substrates (1). Using a variety of imaging modalities, myocardial perfusion under vasodilator stress has been shown to be impaired in patients with HCM (2–4). In the absence of coronary stenoses, this finding is indicative of microvascular dysfunction, but it remains unclear whether the hypoperfusion seen in HCM during stress leads to myocardial tissue deoxygenation and ischemia (5). Furthermore, it is unknown whether HCM mutation carriers without hypertrophy show impaired perfusion and associated deoxygenation during stress.

Blood-oxygen level-dependent (BOLD) cardiovascular magnetic resonance (CMR) or oxygenation-sensitive CMR provides the unprecedented capability to noninvasively assess myocardial tissue oxygenation during vasodilator stress (6–11). BOLD CMR capitalizes on the paramagnetic properties of deoxygenated hemoglobin, which acts as an intrinsic contrast mechanism leading to signal loss in oxygenation-sensitive MR sequences (6,8,10). The change in signal intensity (SI) during vasodilator stress directly reflects myocardial oxygenation status. Oxygenation measurements using BOLD CMR have been shown to be proportional to changes in coronary sinus oxygen saturation (8). As recently shown in patients with coronary artery disease, oxygenation-sensitive CMR can not only identify deoxygenated myocardial segments subtended by stenosed vessels, but also segments with microvascular dysfunction and intermediate SI changes to adenosine stress when compared to normal volunteers (6,7,10). Importantly, myocardial perfusion can be dissociated from oxygenation (i.e., hypoperfusion is not necessarily commensurate with tissue hypoxia). The oxygen demand of the heart muscle may vary in different states, as it may be reduced in hibernating myocardium in line with the down-regulated contractility (12) or may be increased in HCM due to the increased energy cost of contraction (13,14). Thus, compared to perfusion, regional myocardial oxygenation may be a superior parameter reflecting more directly the imbalance between oxygen demand and supply that characterizes ischemia.

The aim of this study was to assess myocardial perfusion and tissue oxygenation during vasodilator stress in patients with overt HCM, as well as in HCM mutation carriers without left ventricular hypertrophy (LVH), and to compare our findings to those in athletes with comparable hypertrophy and normal controls. We hypothesized that tissue oxygenation during stress would be impaired in HCM and that this would not occur in physiological hypertrophy of athletes. If our hypothesis were proven true, it would suggest a central role of stress-induced ischemia, triggering ventricular tachycardia/ ventricular fibrillation, in the pathophysiology of sudden cardiac death in HCM.

## Methods

### Study population

The study was approved by our institutional ethics committee and informed written consent was obtained from each participant. Sixty-eight subjects were recruited into the study: 27 HCM patients with LVH, 10 HCM mutation carriers without LVH, 11 athletes, and 20 normal controls. The diagnosis of HCM was determined on the basis of genetic determination of a pathogenic mutation (11 beta-myosin heavy chain, 11 myosin binding protein C). In the absence of an identified mutation (15 subjects), HCM was defined as the presence of asymmetric LVH (wall thickness ≥15 mm or ≥12 mm in documented familial disease) not originating from other causes. All HCM patients had no cardiovascular risk factors and were recruited from the University of Oxford Cardiomyopathy clinic. Athletes and healthy volunteers had no cardiovascular risk factors or symptoms, no family history of cardiomyopathy or sudden death, and had a normal 12-lead echocardiography (ECG). Athletes were recruited from the City of Oxford rowing club tier 1 team. Only those who performed 6 to 10 h minimum training per week and had maximal wall thickness ≥12 mm by CMR were included in the study. Normal controls were recruited using posters and by word of mouth. In the HCM group, subjects were excluded if they had a blood pressure drop on exercise during a standard Bruce exercise tolerance test or if there was a resting LV outflow tract gradient >30 mm Hg on ECG. Subjects with contraindications to CMR scanning (e.g., pacemakers, defibrillators, claustrophobia) or adenosine (asthma, advanced degree heart block) were not enrolled in the study.

#### CMR Scanning Protocol

CMR was performed on a 3-T system (TIM Trio, Siemens Healthcare, Erlangen, Germany). All participants were instructed to refrain from caffeine in the 24 h preceding the study. Images were acquired with the patient supine, using anterior and posterior phased-array surface coils. For cine CMR, from standard pilot images short-axis cine images covering the entire left ventricle were acquired using a retrospectively ECG-gated balanced steady-state free precession sequence (echo time 1.5 ms, repetition time 3 ms, flip angle 50°). For BOLD-CMR, a set of 2 images was acquired at 3 levels (basal, mid ventricular, and apical) using a T2-prepared ECG-gated balanced steady-state free precession sequence (repetition time/echo time 2.86 ms/1.43 ms, T2 preparation time 40 ms, matrix 168 × 192, field of view 340 × 340 mm, slice thickness 8 mm, flip angle 44°) during peak adenosine stress (140 μg/kg/min) and at rest. Each BOLD image was obtained during a single breath-hold over 6 heartbeats. If necessary, shimming and center frequency adjustments were performed before BOLD imaging, in order to minimize off-resonance artifacts. Immediately following stress BOLD imaging (4 to 5 min after commencing the adenosine infusion), a 0.03 mmol/kg bolus of gadolinium-based contrast (Gadodiamide, Omniscan, GE Healthcare, Oslo, Norway) was injected, followed by 15 ml of saline at a rate of 6 ml/s for first-pass perfusion imaging. During the first-pass of contrast, 3 short-axis images matched in position with the BOLD images were acquired every cardiac cycle using an ECG-gated T1-weighted fast gradient echo sequence (echo time 0.96 ms, repetition time 2 ms, saturation recovery time 95 ms, voxel size 2.1 × 2.6 × 8 mm^3^, flip angle 17°, slice thickness 8 mm). The adenosine infusion was then discontinued and, after a break of at least 25 minutes, another bolus of 0.03 mmol/kg bolus of gadolinium contrast was given for resting perfusion. Patients were instructed to hold their breath as long as possible in end-expiration during perfusion imaging. For late gadolinium enhancement (LGE) CMR, a top-up bolus of 0.06 mmol/kg of Gadodiamide followed by a 15-ml saline flush was administered. After a 5-min delay ECG-gated images were acquired in long- and short-axis planes identical to those of the cine images by using a breath-hold T1-weighted segmented inversion-recovery turbo fast low-angle shot sequence previously described (15). Heart rate and blood pressure were recorded by a vital signs monitor machine at baseline and at 1-min intervals during stress.

### CMR data analysis

For each patient, using commercially available software (Argus version VA60C, Siemens Medical Solutions, Erlangen, Germany), LV volumes, ejection fraction, and mass were calculated by manually tracing the endocardial and epicardial contours in end-diastolic and end-systolic images as previously described (16). End-diastolic wall thickness was measured in 6 basal, 6 midventricular, and 4 apical segments.

Our BOLD analysis method has been published previously (6). Briefly, myocardial SI was measured after manually tracing endocardial and epicardial contours using QMass software (version 6.2.3, Medis, Leiden, the Netherlands). Basal and midventricular short-axis BOLD images were divided into 6 segments (inferior septum, anterior septum, anterior, anterolateral, inferolateral, and inferior), and the apical BOLD slice was divided to 4 segments (septum, anterior, lateral, and inferior) according to American Heart Association 17-segment model (excluding segment 17–true apex) (17). Mean SI were calculated for resting and stress conditions by averaging signal measurements from images during rest and adenosine stress, respectively. BOLD SI measurements were corrected for variations in heart rate between resting and stress and the relative corrected SI change was calculated as previously described (6).

For analysis of myocardial perfusion, SI over time curves were generated by tracing endocardial and epicardial contours (QMass software, Medis, Leiden, the Netherlands) after manual correction for displacement during breathing (18). A region of interest was drawn in the LV blood pool, avoiding any papillary muscles therein, to permit the derivation of an arterial input function. Similar to oxygenation analysis, the myocardium was divided into equiangular segments on the basis of the American Heart Association segmentation model (17). Rest and stress myocardial perfusion up-slopes were calculated using 5-point linear fit model of SI versus time and normalized to the LV blood pool upslope. Myocardial perfusion reserve index (MPRI) was derived for each of the 16 segments, defined as the ratio of stress to rest normalized myocardial perfusion up-slopes.

For LGE analysis, areas of LGE were visually scored as absent or present by consensus of 2 experienced operators (SD 4 years' and TDK 7 years' CMR experience). LGE was considered present only if myocardial enhancement was confirmed on both short-axis and matching long-axis locations. LGE was also quantified, using in house Mc-ROI software (IDL v.6.1, Exelis, Boulder, Colorado), by drawing endocardial and epicardial borders as well as a region of normal intensity myocardium. Hyperenhanced pixels were defined as those with SI >2 standard deviations above the mean of SI in a remote, nonenhanced myocardial region on the same image. The segmental extent of fibrosis was expressed as a percentage of hyperenhanced pixels within each segment.

### Statistical analysis

Data analysis was performed with commercially available software packages (IBM SPSS Statistics, version 19.0, and STATA software version 12.0, Houston, Texas). Values are expressed as mean ± SD unless otherwise stated. The chi-square test or Fisher exact test was used to compare discrete data as appropriate. The methods of linear mixed models were used to analyze perfusion and oxygenation CMR measurements. Measurements within patients are not independent, they are correlated and this was taken into account in the mixed model analyses. The segments and levels are the same across patients and were treated as a fixed effect in the mixed models. Transformation of the data for any variable, MPRI, BOLD, wall thickness, was not necessary to meet the assumptions of the models. Furthermore the small differences in age, sex and body surface area between the 4 groups were taken into account for the perfusion and oxygenation comparisons. The perfusion and oxygenation data for the mixed models are presented as mean ± SE. Analyses of variance with post hoc Bonferroni comparisons were used to compare baseline characteristics and the hemodynamic response to stress between the 4 groups. The homogeneity of variance assumption of analysis of variance was satisfied for all comparisons (Levene's test). Statistical tests were 2-tailed, and a p value of <0.05 was considered to indicate a statistically significant difference.

## Results

### Study population

Subject characteristics are described in Table 1. Perfusion and oxygenation analyses were adjusted for the small differences in age, sex, and body surface area among the 4 groups of subjects. As expected, subjects with overt HCM had significantly increased maximum wall thickness and LV mass index, and smaller LV volumes and higher ejection fractions than normal controls. HCM mutation carriers without hypertrophy had normal cardiac dimensions and LV mass but higher ejection fractions than normal controls. Athletes had increased LV mass and larger end-diastolic LV volumes than controls but similar ejection fractions; although the distribution of hypertrophy was different, as expected, LV mass index was similar in athletes and subjects with overt HCM. Overall, the HCM cohort was considered low risk according to clinical risk stratification (Table 2). During adenosine stress, there were equivalent rises in rate pressure product (normals 78 ± 6%, athletes 65 ± 9%, overt HCM 57 ± 7%, HCM mutation carriers 66 ± 9%; p = 0.18) and heart rate (normals 35 ± 9 beats/min, athletes 30 ± 12 beats/min, overt HCM 30 ± 15 beats/min, HCM mutation carriers 32 ± 9 beats/min; p = 0.58) in all groups.

### Changes in myocardial perfusion under adenosine stress

**Figure 1:**
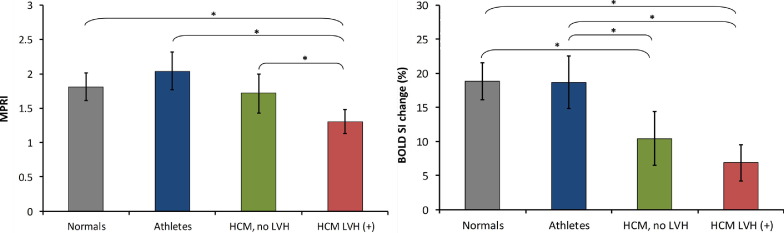
Oxygenation and Perfusion Changes in the 4 Groups Bar graph showing perfusion (myocardial perfusion reserve index, **left**) and oxygenation changes (measured as blood oxygen level-dependent signal intensity change, **right**) in normals, athletes, hypertrophic cardiomyopathy (HCM) mutation carriers without left ventricular hypertrophy (LVH), and patients with overt HCM. **Error bars** represent the 95% confidence intervals for the mean. *p < 0.05.

Confirming previous CMR and positron emission tomography (PET) studies (2–4), myocardial perfusion reserve was significantly reduced in HCM patients with LVH compared to healthy controls (MPRI in subjects with HCM 1.3 ± 0.1 vs. 1.8 ± 0.1 in normals; p < 0.0001) (Fig. 1). Furthermore, comparing physiologic to pathologic hypertrophy, there was a significant reduction in MPRI in patients with HCM compared to athletes (1.3 ± 0.1 vs. 2.0 ± 0.1; p < 0.0001). In contrast, we now report that myocardial perfusion reserve was preserved in HCM mutation carriers without hypertrophy (1.7 ± 0.1; p = 0.61) compared to normals, and significantly higher than in overt HCM (1.3 ± 0.1; p = 0.02). Athletes (2.0 ± 0.1), normals (1.8 ± 0.1; p = 0.11 vs. athletes), and HCM mutation carriers without LVH (1.7 ± 0.1; p = 0.11 vs. athletes) all had similar myocardial perfusion reserve.

### Changes in myocardial oxygenation under adenosine stress

**Figure 2:**
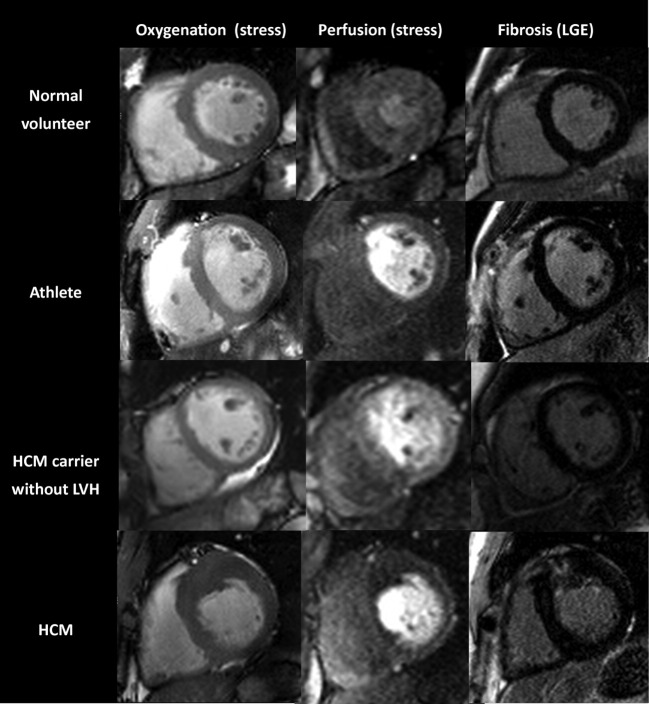
Cardiovascular Magnetic Resonance Perfusion, Oxygenation, and Late Gadolinium Enhancement Examples Oxygenation, perfusion, and corresponding late gadolinium enhancement images in the 4 groups. Note the asymmetric anteroseptal hypertrophy in the HCM patient, which was associated with patchy fibrosis. Perfusion reserve (mean myocardial perfusion reserve index values ranging from 1.1 to 1.8) and oxygenation signal intensity change (ranging from 3% to 13%) were impaired. Abbreviations as in Table 1.

During vasodilator stress, subjects with overt HCM showed evidence of blunted oxygenation response (SI change 6.9 ± 1.4%) compared to normal controls (18.9 ± 1.4%; p < 0.0001) and athletes (18.7 ± 2.0%; p = 0.0005) (Fig. 1). Remarkably, HCM mutation carriers without hypertrophy had a similarly blunted oxygenation response to adenosine stress as subjects with overt HCM (SI change in HCM mutation carriers without LVH 10.4 ± 2.0% vs. HCM patients with LVH 6.9 ± 1.4%; p = 0.16) (Fig. 1). In contrast, athletes showed a normal oxygenation response during stress, which was not different from controls (SI change 18.9 ± 1.4% in normal controls and 18.7 ± 2.0% in athletes; p = 0.95). Stress oxygenation changes mirrored changes in perfusion reserve in healthy controls, athletes, and patients with HCM. In contrast, in HCM mutation carriers, there was dissociation between perfusion and oxygenation measurements (i.e., a blunted oxygenation response despite preserved perfusion reserve). Figure 2 shows representative CMR images of oxygenation, perfusion, and LGE.

### Relationship among oxygenation, perfusion, and fibrosis in HCM

In patients with HCM, 229 of 432 segments (53%) showed patchy fibrosis on LGE imaging, and the extent of segmental enhancement ranged from 2% to 100% (median 30.2%; 95% confidence interval: 27.6 to 34.2). Overall, 18 of 27 (67%) patients with overt HCM had positive LGE and the mean quantity of scar was 32.3 ± 10.7 g. None of the controls, athletes, and HCM mutation carriers had evidence of fibrosis on LGE imaging. In line with previous studies (4), we found that in HCM (including both mutation carriers without LVH and patients with overt HCM), segments with fibrosis had a significantly lower MPRI (1.2 ± 0.04) compared to segments without fibrosis (1.5 ± 0.03, p < 0.001). A novel finding of this study is that fibrosis in HCM was also associated with a greater blunting of myocardial oxygenation during vasodilator stress. BOLD SI increase was less in segments with fibrosis (6.2 ± 0.7%) compared to segments without fibrosis (10.1 ± 0.7%; p < 0.001).

### Relationship between oxygenation, perfusion, and wall thickness

**Figure 3:**
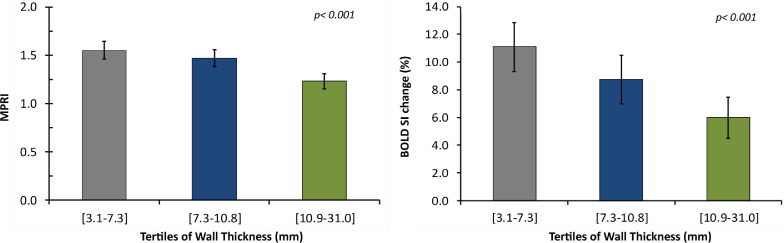
Oxygenation and Perfusion Measurements Across Tertiles of Wall Thickness in HCM Within the HCM group (including mutation carriers without hypertrophy), there is a stepwise decrease in perfusion **(left)** and oxygenation **(right)** measurements across tertiles of end-diastolic wall thickness. **Error bars** represent the 95% confidence intervals for mean. Abbreviations as in Table 1.

**Figure 4:**
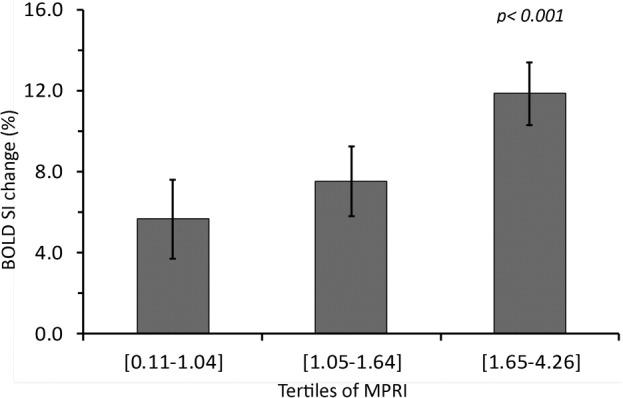
Oxygenation Measurements Across Tertiles of Myocardial Perfusion Reserve Index in HCM There is a stepwise increase in oxygenation measurements across tertiles of myocardial perfusion reserve index in the combined HCM group (including mutation carriers without hypertrophy and patients with overt HCM). **Error bars** represent the 95% confidence intervals for mean. Abbreviations as in Table 1.

In the combined HCM group (including both HCM mutation carriers without hypertrophy and subjects with overt HCM), perfusion reserve measurements were found to decrease significantly across tertiles of end-diastolic wall thickness (Fig. 3), confirming previous studies (4). Stress oxygenation measurements showed a similar behavior (i.e., decreased significantly across tertiles of end-diastolic wall thickness) (Fig. 3); however, in contrast with perfusion, oxygenation values in all 3 tertiles were abnormal. Furthermore, there was a significant association between oxygenation and perfusion measurements with a stepwise increase in BOLD SI measurements across tertiles of MPRI (Fig. 4).

## Discussion

This study examined myocardial oxygenation during vasodilator stress, together with myocardial perfusion reserve, in patients with overt HCM and in HCM mutation carriers without hypertrophy, in comparison with control subjects and athletes with physiologic cardiac hypertrophy.

### Perfusion and oxygenation in HCM

Previous studies, mostly using PET-perfusion measurements but also first-pass perfusion CMR, have shown that HCM patients have reduced hyperemic myocardial blood flow during vasodilator stress compared to healthy controls (2–4). Whether such impairment in stress perfusion levels is severe enough to translate into limited myocardial oxygen supply and, thus, tissue ischemia, has not been known. Myocardial hypoperfusion cannot necessarily be equated with hypoxia (6), given that the oxygen demand of the myocardium may vary and may be increased in HCM, where myocardial contractility is more energy costly (13,14), or may be reduced in hibernating myocardium, where oxygen demand is down-regulated (12). Thus, oxygenation assessment is a much more direct measure of myocardial tissue ischemia than surrogates such as perfusion or contractile abnormalities during stress. Our study unequivocally shows that both oxygenation and perfusion are significantly impaired during vasodilator stress in patients with overt HCM, and that oxygenation and perfusion changes are correlated (Fig. 4). In addition, we show that the degree of impairment of oxygenation and perfusion relates to the extent of hypertrophy (Fig. 3), suggesting that the most hypertrophied segments are most prone to stress-induced tissue ischemia. Furthermore, myocardial segments with patchy fibrosis were found to have a greater reduction in oxygenation and perfusion compared to segments without fibrosis. Fibrotic tissue itself has low oxygen demand, but its presence in a segment also suggests that, in that region, myocytes and microvessels are more severely affected by the disease, thus showing greater perfusion and oxygenation impairment.

### Perfusion and oxygenation in HCM mutation carriers

The most intriguing finding of our study comes from the cohort of HCM mutation carriers without LVH. Interestingly, we describe, for the first time, that such subjects do not show impaired myocardial perfusion reserve, but despite that, they still demonstrate an abnormal oxygenation response during stress. This observation is most likely explained by the fact that sarcomere-gene mutations increase the energy cost of contraction, even before the onset of hypertrophy (14), and that this may lead to disproportionately increased oxygen demand during stress, resulting in tissue deoxygenation. Therefore, the abnormal oxygenation changes observed in overt HCM patients in this study likely reflect both the increased demand and the microcirculatory changes.

### Identification of HCM mutation carriers before the onset of hypertrophy

Recent studies have demonstrated the value of CMR and advanced echocardiography in characterizing HCM mutation carriers without LVH. Increased LV torsion (19), reduced segmental peak circumferential and longitudinal strain (20,21) are functional LV abnormalities that have been proposed for the early identification of HCM carriers. Our results suggest that BOLD CMR is also promising in this respect. However, it is unknown whether diastolic dysfunction or impaired oxygenation during stress represents a marker for the future evolution of LVH, cardiac symptoms, or major cardiac events. Further longitudinal large-scale studies should investigate these intriguing possibilities.

### Physiologic versus pathologic hypertrophy

Our study shows that, in contrast to HCM patients with pathologic hypertrophy, athletes with a similar increase in LV mass but physiologic hypertrophy show neither perfusion nor oxygenation limitations during vasodilator stress, demonstrating that the athlete's heart is fully adapted under these conditions. Previous studies that assessed myocardial perfusion in elite athletes using PET or echocardiography have yielded discrepant results. Some studies found an increase in coronary flow reserve in athletes as compared to normal volunteers (22,23). In contrast, Kalliokoski et al. observed no difference in resting or adenosine-stimulated myocardial perfusion between elite athletes and sedentary healthy individuals (24).

### Clinical implications

The current study provides important insights into the pathophysiology of ischemia in HCM and HCM gene carriers. Sudden cardiac death during stress is a dreaded complication of HCM. Our study suggests a potential mechanism for this. We speculate that stress-induced myocardial deoxygenation would lead to reduction in the cardiomyocyte phosphorylation potential, impairing the function of SERCA (the energetically most demanding enzyme involved in cardiac contraction) (25), subsequently leading to intracellular Ca^++^ overload, thus triggering ventricular tachycardia/ventricular fibrillation. Interestingly, sudden death can also occur in a subset of HCM patients with no or minimal LVH, and it is notable that our study showed that a blunted oxygenation response occurs even in HCM mutation carriers without detectable LVH. Further studies will have to investigate these intriguing possibilities. We also plan to extend our studies to include analysis of the oxygenation response in the various specific disease-genes underlying HCM.

### Study limitations

We excluded HCM patients at high risk for sudden death and, therefore, there is some selection bias in our study. However, this may also be viewed as a strength of the study, suggesting a role of oxygenation-sensitive CMR in the risk assessment of patients traditionally considered lower risk. The athletes group was younger than the HCM subjects. However, in absolute terms, the difference was relatively small, it is difficult to find elite athletes older than 40 years, and any differences in age, sex, or body surface area among the 4 groups were taken into account in our statistical analyses. We believe that this did not affect our oxygenation and perfusion results as it is well known that myocardial blood flow declines with aging only after the age of 60 years (26,27). Finally, our sample size is relatively small, particularly for the HCM carriers group without LVH, and confirmation of our findings in large-scale studies is needed.

## Conclusions

In overt HCM, both perfusion and oxygenation are impaired during vasodilator stress. However, in HCM mutation carriers without LVH, only oxygenation is impaired. In physiologic hypertrophy (athlete's heart), stress perfusion and oxygenation are normal. We speculate that deoxygenation is a potential mechanism of stress-induced sudden cardiac death in HCM. Finally, BOLD CMR assessment of myocardial oxygenation may become a useful test in HCM, as potential novel risk factor, and to aid the differential diagnosis of HCM, HCM mutation carriers, and athlete's heart.

## Figures and Tables

**Table 1:** Baseline Characteristics of Study Groups

	Normal Controls (n = 20)	Athletes (n = 11)	HCM Mutation Carriers (No LVH) (n = 10)	HCM Patients (With LVH) (n = 27)
Age, yrs	39.6 ± 9.8	31.5 ± 9.3	36.2 ± 14.2	48.2 ± 9.2⁎†‡
Male	13 (65)	8 (73)	4 (40)⁎†§	19 (70)
BSA, m^2^	1.93 ± 0.19	1.88 ± 0.17	1.71 ± 0.21⁎§	2.04 ± 0.22
LV end-diastolic volume index, ml/m^2^	78.5 ± 15.3	92.2 ± 19.9	63.3 ± 8.9⁎†	58.6 ± 12.2⁎
LV end-systolic volume index, ml/m^2^	30.0 ± 7.2	33.8 ± 12.8	17.4 ± 6.3⁎†	16.2 ± 5.4⁎†
LV ejection fraction, %	61.9 ± 4.1	64.9 ± 6.1	72.9 ± 7.8⁎†	73.6 ± 5.3⁎†
LV mass index, g/m^2^	64.7 ± 9.9	92.8 ± 16.1⁎‡	60.4 ± 10.0	95.5 ± 40.2⁎‡
Maximal LV wall thickness	10.1 ± 1.3	12.4 ± 0.7	9.6 ± 0.9	19.3 ± 5.4⁎†‡
Rest heart rate, beats/min	55.6 ± 7.8	54.0 ± 8.6	60.3 ± 10.2	57.4 ± 8.9
Stress heart rate, beats/min	90.4 ± 10.7	87.4 ± 17.6	92.3 ± 14.5	87.4 ± 17.6

Values are mean ± SD or n (%). BSA = body surface area; LV = left ventricular.

⁎ p < 0.05 versus normal controls.

† p < 0.05 versus athletes.

‡ p < 0.05 versus hypertrophic cardiomyopathy (HCM) mutation carriers without hypertrophy.

§ p < 0.05 versus HCM patients with left ventricular hypertrophy (LVH).

**Table 2:** Clinical Risk Stratification and Medications in HCM Cohort

	HCM Mutation Carriers Without LVH (n = 10)	HCM Patients With LVH (n = 27)
No. of SCD risk factors		
0	8 (80)	15 (55)
1	1 (10)	8 (30)
2	1 (10)	4 (15)
3	0 (0)	0 (0)
Family history of SCD	2 (20)	11 (41)
Unexplained syncope	0 (0)	1 (4)
NSVT on Holter monitor	1 (10)	3 (11)
Abnormal exercise blood pressure response	0 (0)	0 (0)
Maximum LV wall thickness ≥30 mm	0 (0)	1 (4)
LV outflow tract gradient	0 (0)	0 (0)
Beta-blockers	4 (40)	18 (67)
Calcium-channel blockers	0 (0)	22 (6)

Values are n (%). NSVT = nonsustained ventricular tachycardia; SCD = sudden cardiac death; other abbreviations as in Table 1.
